# Supplementary figures and images for: Image-based parameter inference for epithelial mechanics
Source: PLoS Comput Biol. 2022 Jun 23;18(6):e1010209. doi: 10.1371/journal.pcbi.1010209 (PMC9223404; doi:10.1371/journal.pcbi.1010209)

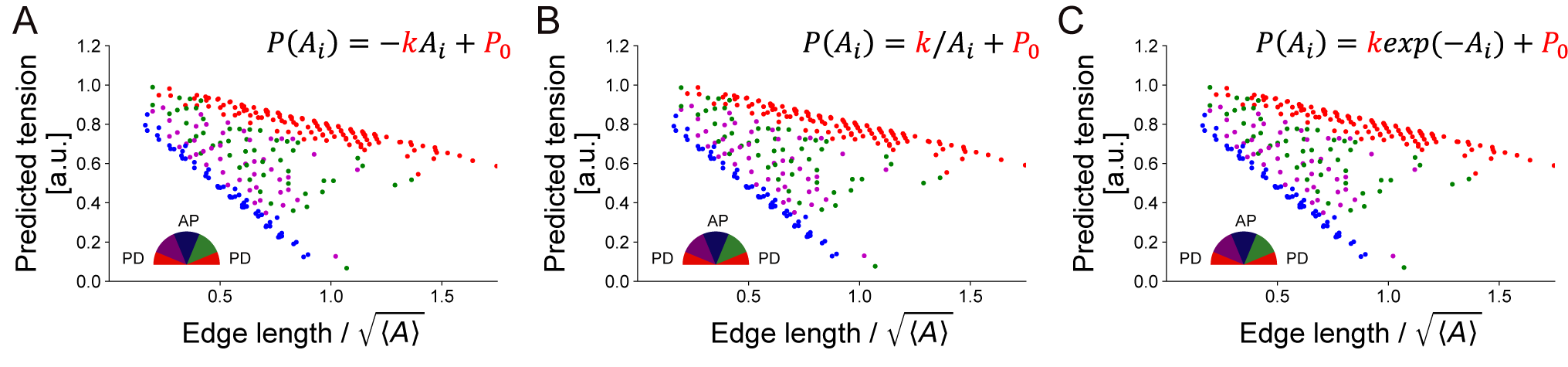

Supplement: S1 Fig — (A–C) Tension predicted by estimated parameters is plotted against junction length. Dot color indicates the orientation of each junction relative to the PD axis of the wing (semicircle). Pressure functions used for estimation were P(Ai) = −kAi+P0 (A), P(Ai) = k/Ai+P0 (B), and P(Ai) = kexp(−Ai)+P0 (C). The plot in (A) is the same as that shown in Fig 1N. (TIF) [file pcbi.1010209.s001.tif]

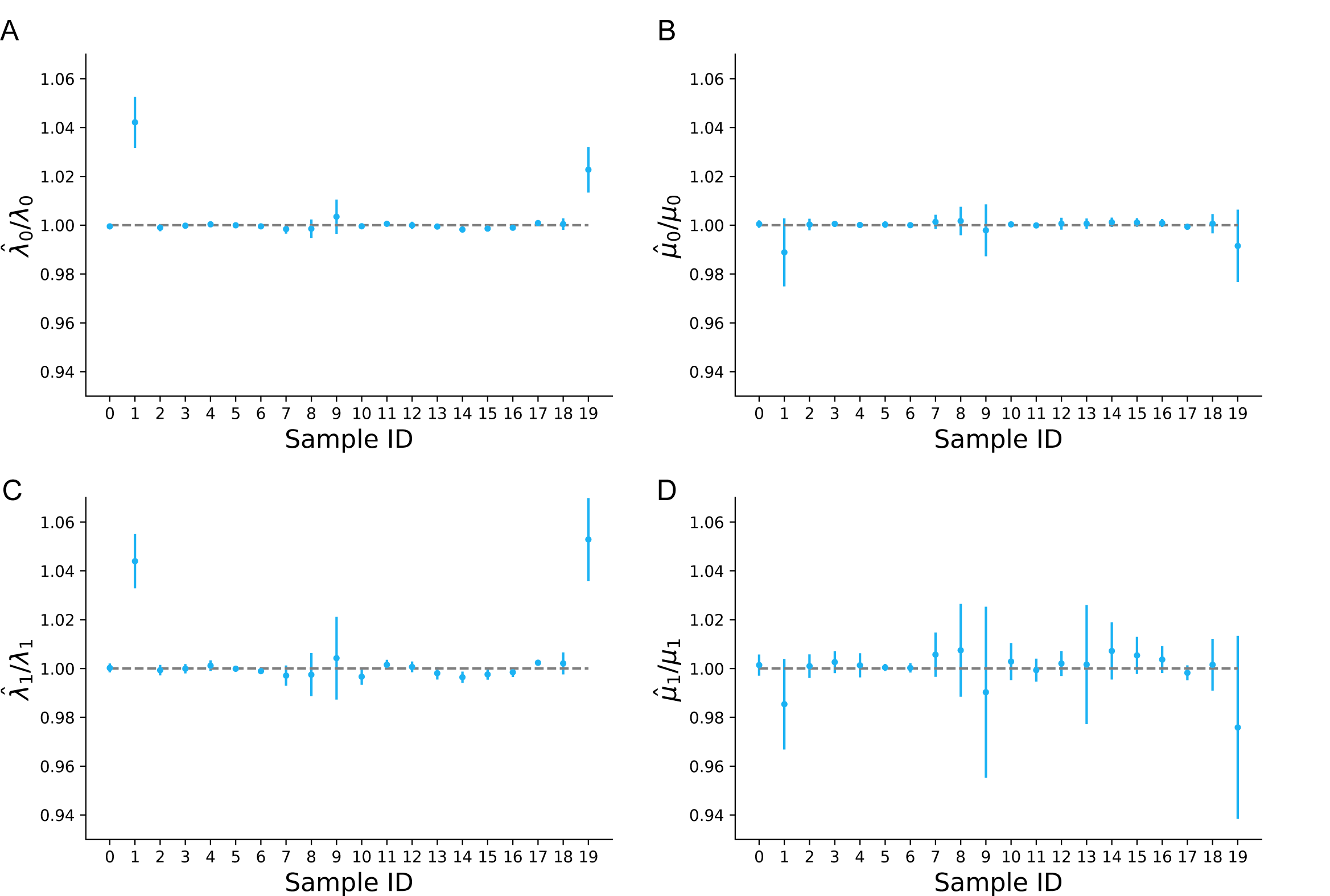

Supplement: S2 Fig — (A–D) The uncertainty quantification for λ0 (A; the line tension), μ0 (B; the anisotropy in the line tension), λ1 (C; the spring constant of junction), and μ1 (D; the anisotropy in the spring constant of junction). Synthetic data were generated from 20 different parameter sets. The standard error of the parameter was calculated from the residue and normalized by considering the error propagation based on the relationship described in the legend of Fig 3. Dots indicate the estimated value of the parameter. The error bar is defined as [q^−1.96σ^q,q^+1.96σ^q], where q^ and σ^q are the estimated values of the normalized parameter and its standard error, respectively. The data showed that the error bars were narrow and included the true values of μ0 and μ1 in all samples and those of λ0 and λ1 in 12 of the samples. (TIF) [file pcbi.1010209.s002.tif]

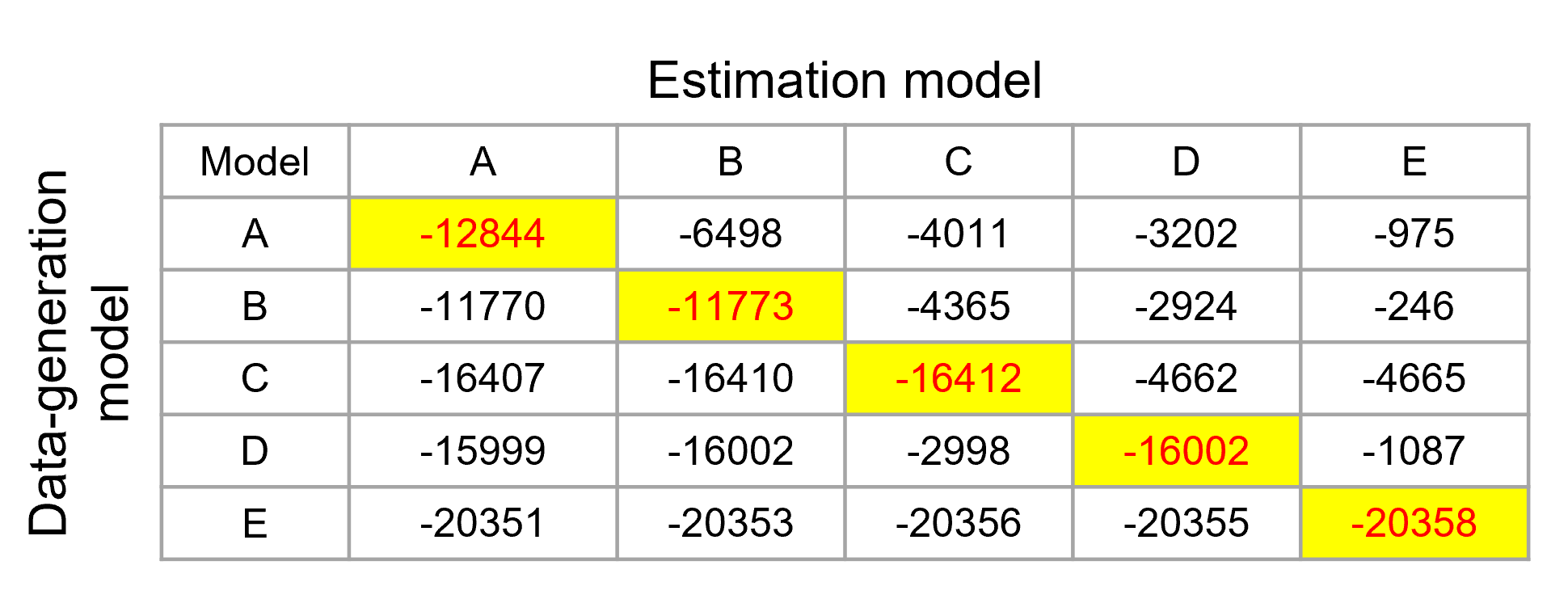

Supplement: S3 Fig — (A) Table of AIC values in synthetic data set. Synthetic data were generated using different models (data generation models), and AIC values were calculated for each model (estimation models). In each row, AIC values take the minimum value when the data generation model is used for inference (highlighted in yellow). (TIF) [file pcbi.1010209.s003.tif]

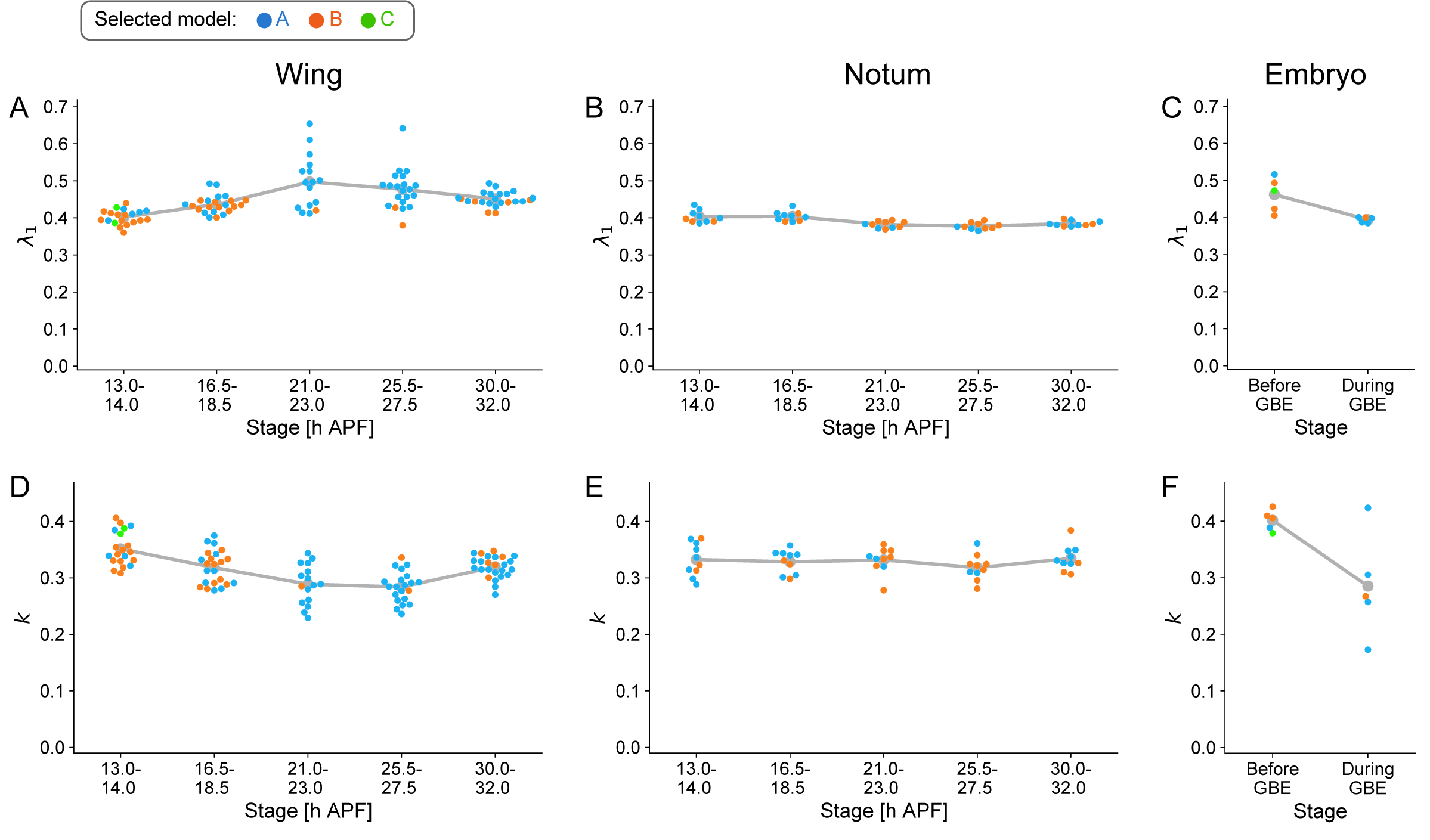

Supplement: S4 Fig — (A–F) Estimated values of λ1 (A–C; the spring constant of junction) and k (D–F; the elastic modulus of cells) at the stage indicated in the pupal wing (A, D), pupal notum (B, E), and embryo (C, F). The gray line connects the average estimated values of parameters for each stage. Dot colors represent a model selected by AIC. (TIF) [file pcbi.1010209.s004.tif]
