# Supplementary material for: Image-based parameter inference for epithelial mechanics
Source: PLoS Comput Biol. 2022 Jun 23;18(6):e1010209. doi: 10.1371/journal.pcbi.1010209 (PMC9223404; doi:10.1371/journal.pcbi.1010209)
Supplement: Attachment — Submitted filename: Ogitarr-Response.pdf [file pcbi.1010209.s005.pdf]

Revision : PCOMPBIOL-D-21-01805

Image-based parameter inference for epithelial mechanics

by Goshi Ogita, Takefumi Kondo, Keisuke Ikawa, Tadashi Uemura, Shuji Ishihara, Kaoru Sugimura

Response to Reviewers

We would like to thank the reviewers for their insightful and constructive comments. We believe the manuscript has improved significantly as a result of the comments and points raised by the reviewers. In particular, we:

- Revised the manuscript to improve readability.
- Extended the synthetic data study to evaluate if the confidence intervals trap the true parameter value and if the parameter estimation is affected by errors in input data.
- Uploaded the code for parameter inference and model selection to GitHub.

In addition, we have added a new Supplementary Fig. 1, which shows that the choise of pressure functions does not affect the predicted tensions.

The figure legends and relevant sections were changed accordingly. The revised points are typed in blue in the revised manuscript. Our detailed point-by-point responses are provided below.

Answers to Reviewer 1

In their manuscript "Image-based parameter inference for epithelial mechanics," the authors formulated an image-based statistical method for estimating mechanical parameters of epithelial cellular networks. Then, they investigated candidate mechanical models by substituting the model functions into the force-balance equations and estimating model parameters with least-squares optimization. They verified the approach with synthetic data and applied the method to *Drosophila* epithelial tissues. The method identifies patterns in tension anisotropy correlated with extrinsic forces applied to the pupal wing and gene patterning within the embryo. The main finding of the work is inference of a more complex relationship between tension and cell-cell boundary lengths and angles. The method provides robust estimates of mechanical properties that can potentially be used for other contexts of epithelial morphogenesis. Their model selection approach has the potential to inform our understanding of the biophysical regulation of tissue morphogenesis.

We are grateful to the reviewer for these encouraging comments. Point-by-point responses to his/her comments are given below.

Major comments

1) In general the paper is clear and concisely written. Occasionally, the dense use of parameters detracts from readability. This could be ameliorated by reminding the reader of the physical interpretation of the parameters in each separate section and figure caption.

We followed the reviewer's suggestion and added the definitions of parameters in each separate section and figure caption.

2) Readers who are not *Drosophila* wing experts would benefit from having Fig. S1 incorporated into the main text

We moved Supplementary Fig. 1A–F to the main text (new Fig. 2).

3) In the authors' summary, it is claimed that the proposed methods give "more accurate estimates" than previous methods, but a systematic test of this claim is not provided. This could be more explicitly shown.

We thank the reviewer for their constructive comment. For synthetic data generated by the conventional model with a typical parameter set $(\Lambda_0, \Gamma) = (0.12, 0.04)$, the estimation errors of Λ_0 and Γ were 0.27 % and -0.20 %, respectively (ll. 280–283 in the original manuscript and ll. 308–311 in the revised manuscript). However, an Approximate Bayesian Computation (ABC)–based method was shown to have estimation errors larger than 6 % for the same parameter set (Kursawe et al. *J. Theor. Biol.* (2018)). This indicated that our method resulted in more accurate estimates than the previous method under these conditions. As suggested by the reviewer, to validate this statement, a systematic comparison was required between our method and previous methods. Regrettably, the ABC–based method required 10^6 numerical simulations for estimating two parameters (Kursawe et al. *J. Theor. Biol.* (2018)), thus the deadline for the submission of the revised manuscript would not

be met if the comparison was to be performed. We, therefore, deleted the sentence from the authors' summary (ll. 44–45).

4) It would be very helpful for the community and the impact of the paper if the authors would publish their code on Github. It would be very helpful for transparency and verification (and to increase the potential impact of the work). Sharing the method/ code as an application would greatly enhance the perceived value to the field.

We have uploaded the code for parameter inference and model selection to Github (https://github.com/Sugimuralab/ImageBasedParameterInferenceForEpithelialMechanics).

Minor typos:
Line 161: Add indefinite article: "In [a] tissue"
Line 388-389: Clarify: "same in both [classes] of models [non-conventional and conventional""

We revised the text according to the reviewer's comments (ll. 127 and ll. 416–417).

Answers to Reviewer 2

Ogita et al. present a simple approach to estimating the mechanical properties of cells and tissues from image data. This approach is applicable to both in vitro and in vivo systems.

The approach presented is essentially a two stage estimation approach:

- First, forces and cell shapes are related via a previously established 'Bayesian force/stress inference' approach.

- Second, candidate constitutive equations are substituted into the above and parameters estimated via simple regression. Nested models can also be compared via e.g. AIC.

We thank the reviewer for his/her careful reading and suggestions, which have strengthened this study.

#General comments:
1) I think this is a generally sensible approach. It relies heavily on the first stage 'Bayesian force/stress inference' method developed by two of the authors. The second stage is, as the authors note, essentially just linear/nonlinear regression.
My only real concern is that it is somewhat pushing it to call this a 'new statistical method', given the new, additional stage is simply regression. However, again, this is still a perfectly reasonable approach. I'm glad the authors include a synthetic data study.

We agree with the reviewer and thus edited the manuscript to address this concern (*e.g.*, ll. 22–23). Please see our response to minor issue 5.

Minor issues
1) It would be good to include some measurement error in the synthetic data study. How robust are the results to this?

Following the reviewer's suggestion, we quantified the deviation of the estimated parameter values on the addition of noise to the vertex coordinates in the synthetic data as performed for the *in vivo* data (ll. 321–334; new Fig. 4A, E, I, M). The deviation of the estimated parameter values in noised data sets was within 25 % in most cases.

2) Relatedly, regression methods allow simple confidence/credible intervals to be formed (taking the first stage as given, perhaps). It would be good to evaluate the performance of these on the synthetic data. E.g. given some measurement/imaging error, how often do the intervals trap the true parameters?

We calculated the 95 % confidence intervals for the estimated parameters of the synthetic data set. The data indicated that the 95 % confidence intervals were narrow and trapped the true values in several cases. The results were shown in a new Supplementary Fig. 2 (ll. 307–308).

3) The organisation of the article could perhaps be improved by combining 'Experimental systems', 'A mechanical parameter estimation method' and 'Materials and methods'. These all seem like 'Methods' to me, and I found the division a bit confusing. It would also help to e.g. present the synthetic experimental system directly followed by the 'real' experimental

systems within the same section. E.g. have 'Experimental systems' as part of 'Materials and Methods' and with 'Synthetic', 'Pupal wing', Pupal not' and 'Embryo' as subheadings.

The 'Experimental systems' section was removed and a new 'Data sets' section was created. The data set included the synthetic and *in vivo* data sets used in this study (ll. 237–294).

4) Line 164. I would delete 'uniquely' as other methods might exist.

We revised the text according to the reviewer's comment (ll. 130).

5) Line 399. 'We developed a new' statistical method to' -> We developed a simple, two-stage statistical approach to'

We appreciate the suggestion made by the reviewer. The sentence was replaced with the sentence suggested by the reviewer (ll. 428–429).
