# Supplementary material for: Image-based parameter inference for epithelial mechanics
Source: PLoS Comput Biol. 2022 Jun 23;18(6):e1010209. doi: 10.1371/journal.pcbi.1010209 (PMC9223404; doi:10.1371/journal.pcbi.1010209)
Supplement: Attachment — Submitted filename: Ogitarrr-Response.pdf [file pcbi.1010209.s006.pdf]

Revision : PCOMPBIOL-D-21-01805R1

Image-based parameter inference for epithelial mechanics

by Goshi Ogita, Takefumi Kondo, Keisuke Ikawa, Tadashi Uemura, Shuji Ishihara, Kaoru Sugimura

Answers to Reviewer 1

Reviewer #1: The revised version largely addressed issues raised in the first review. A couple of remaining curiosities in the manuscript could be addressed in the final version:

We thank the reviewer for his/her careful reading and suggestions, which have strengthened this study. Point-by-point responses to his/her comments are given below.

1. The manuscript appears to be methodologically sound but the linked Github only provides parameter estimation code, not force inference. It could be more clearly described to enable full verification.

We have uploaded the code for Bayesian force inference to Github (https://github.com/IshiharaLab/BayesianForceInference). This URL is cited in https://github.com/Sugimuralab/ImageBasedParameterInferenceForEpithelialMechanics.

2. While several genotypes such as sqhp-sqh-GFP were reported in the study, it was not clear that sqh was quantified. Several claims about accumulation were stated, but it was not clear that this was shown.

Studies by others and us have reported the quantification of the myo-II localization in *Drosophila* wing, notum, and germband (refs. 15, 22, 36–39, 46 among others). For instance, we performed the quantification of myo-II in control and hinge-severed wings and showed that wing cells localize myo-II to resist the stretching force from the hinge (ref. 46). The increase in myo-II level during junction shrinkage, which is represented in the model equations considered in this study, has been quantified in *Drosophila* wing and embryo and reported in Fig. 1 in ref. 37, Fig. 3 in ref. 38, and Fig. 5 in ref. 39. We confirmed the claim ourselves in a recent study (Ikawa, Ishihara, Tamori, Sugimura. *In revision*). To address the reviewer's concern, we have revised the text (ll. 161).

Additionally, ref. 66 did not seem to use the nomenclature sqhp and this should be defined.

Ref. 66 reports the following: "We initially constructed both the N-terminal (gfp-sqh) and the C-terminal (sqh-gfp) versions of the chimera, both under the control of the natural sqh promoter." We have revised the text in the Materials and Methods section to include the information reported in ref. 66 (ll. 575–576).

Biological significance of findings could be highlighted better. There is a lack of quantification of Myosin, which could be clarified.
3. A summary figure S5 is helpful in understanding the main biological inferences made from

the study. Burying it in the SI will obscure the key findings. The meaning of the color of the arrows in the right panel could be clarified.

We have followed the reviewer's suggestion and moved Supplementary Fig. 5 to the main text (new Fig. 7; Some of the panels were moved to Fig. 2). Regarding the quantification of Myosin, please see our response to point 2 above.

The color of arrows in the right panel had been defined in the legend as "line color represents junctions along the specific axis of the tissue and the arrow width represents the magnitude of the feedback" (ll. 922–924 in the main text of the revised manuscript and ll. 53–55 in the supplementary information of the original manuscript). To further clarify this point, we have revised the new Fig. 7.

Answers to Reviewer 2

Reviewer #2: The authors have essentially addressed all my comments and the article is a useful contribution to the literature.

We appreciate his/her positive evaluation on the significance of this study.

However I would note that the comments like
> 95% confidence intervals included the true value in most cases (Supplementary Fig. 2).
are not quite precise. In particular, if an interval procedure does not generate a close to 95% coverage rate in a synthetic experiment with sufficiently many repetitions then it can't really be called a 95% confidence interval in the frequentist sense -- 95% coverage being the defining property of a 95% confidence interval. However, it is well known that many approximate confidence interval procedures don't exactly satisfy their intended (or nominal) coverage. This is a long way of saying that it would be better to say e.g.
> The approximate 95% confidence intervals included the true value in most cases (Supplementary Fig. 2).

Finally, I'm wondering if any noise was added during the synthetic data experiment -- the intervals are very narrow. If there was and the noise only had a very small impact on the estimates that's fine, but surprising. If no noise was added then I'm wondering how 95% confidence intervals in the frequentist sense (i.e. based on a sampling distribution under a noise model) were formed. Perhaps these are more like Bayesian credible intervals?

We thank the reviewer for raising this important point. Since neither observation nor system error was included in the synthetic data used in Supplementary Fig. 2, the interval quantified is more like credible intervals as the reviewer pointed out. In the revised manuscript, we have added the procedure for the calculation of the error bars in Supplementary Fig. 2 in its legend. We avoided using the confidence/credible interval in the legend of Supplementary Fig. 2 and in the main text (ll. 308–309). We would like to defer the evaluation of credible intervals to our future study, in which we implement the parameter estimation method in a framework of Bayesian statistics (Ogita, Xin, Ishihara, Sugimura. *Unpublished*).

Most of this is nit-picking based on supplementary material, but I think being a bit more precise in how these things are discussed would increase the chance that the method can be appropriately used by others on real-world problems.

We are grateful to the reviewer for his/her constructive comments throughout the review process of the manuscript.
